# A Label-Free Fluorescent Aptasensor for Detection of Staphylococcal Enterotoxin A Based on Aptamer-Functionalized Silver Nanoclusters

**DOI:** 10.3390/polym12010152

**Published:** 2020-01-07

**Authors:** Xueyan Zhang, Imran Mahmood Khan, Hua Ji, Zhouping Wang, Huili Tian, Wenbo Cao, Weiyu Mi

**Affiliations:** 1 School of Food Science and Technology, Shihezi University, Shihezi 832000, China; zxy18139260082@163.com (X.Z.);; 2 State Key Laboratory of Food Science and Technology, Jiangnan University, Wuxi 214122, China; 3 School of Food Science and Technology, Jiangnan University, Wuxi 214122, China; 4 Collaborative Innovation Center of Food Safety and Quality Control, Jiangnan University, Wuxi 214122, China; 5 International Joint Laboratory on Food Safety, Jiangnan University, Wuxi 214122, China

**Keywords:** staphylococcal enterotoxin A (SEA), aptamer-functionalized AgNCs, polypyrrole nanoparticles (PPyNPs), fluorescence aptasensor detection

## Abstract

Staphylococcal enterotoxin A (SEA) is a worldwide public health problem accounting for the majority of food poisoning which is produced by *Staphylococcus aureus*, threatening human health and leading to various foodborne diseases. Therefore, it is of great significance to develop a sensitive detection method for SEA to ensure food safety and prevent foodborne diseases in humans. In this study, an adaptive fluorescence biosensor for the detection of staphylococcal enterotoxin A (SEA) was designed and developed by combining DNA silver nanoclusters (DNA-AgNCs) with polypyrrole nanoparticles (PPyNPs). Fluorescent AgNCs, synthesized using aptamers as templates, were used as fluorescence probes, whose fluorescence was quenched by PPyNPs. In the presence of the target SEA, DNA-AgNCs were forced to desorb from the surface of PPyNPs through the binding of SEA with the aptamer-DNA-AgNCs, thereby resulting in fluorescence recovery. Under the optimized conditions, the relative fluorescence intensity (FI) showed a linear relationship with the SEA concentration in the range from 0.5 to 1000 ng/mL (*Y* = 1.4917*X* + 0.9100, *R*^2^ = 0.9948) with a limit of detection (LOD) of 0.3393 ng/mL. The sensor was successfully used to evaluate the content of SEA in milk samples, and the recovery efficiency of SEA was between 87.70% and 94.65%. Thus, the sensor shows great potential for application in food analysis. In short, the proposed platform consisted of an aptamer fluorescent sensor that can be used for the ultrasensitive detection of various toxins by taking advantage of the excellent affinity and specificity of corresponding aptamers.

## 1. Introduction

Food safety is a major global public health concern, especially the hazards caused by foodborne pathogens, among which *Staphylococcus aureus*, a Gram-positive bacteria that can produce a variety of enterotoxins, neurotoxins, and cytotoxins, is one of the most common pathogens [1,2,3]. There are currently 24 reported species of *Staphylococcus aureus* enterotoxin [4]. Among them, staphylococcal enterotoxin A (SEA) is responsible for the main cases of *Staphylococcus aureus* food poisoning worldwide [5,6]. It often contaminates the foods such as meat, poultry, eggs, milk, and dairy products [5,6,7,8,9,10]. Moreover, the intake of SEA up to 50 ng (body weights of 70 kg) [11] can cause food poisoning and other diseases, as well as the exacerbation of various diseases [12,13]. For instance, it is also likely to cause life-threatening toxic shock [14,15]. Additionally, the cooking process may not eliminate the toxin in the contaminated food because of heat-resistant [13], proteolytically stable, and small monomeric proteins (molecular weight (MW) = 27 kDa) of SEA that can resist a series of harsh conditions [16].

Therefore, rapid detection of SEA is essential to ensure food safety. In this regard, many fast, highly sensitive, and specific SEA detection methods were developed in recent years, as shown in Table S2 (Supplementary Materials), such as high-performance liquid chromatography (HPLC) methods [17] and liquid chromatography–tandem mass spectrometry (LC–MS/MS) [18,19]; however, these methods require expensive equipment and reagents, and they take more time to prepare samples and perform analysis. In addition, various biosensors such as quartz crystal microbalances [20], electrochemical immunosensors [21,22], enzyme-linked immunosorbent assays [23], chemiluminescence enzyme immunoassay (CLEIA) [24], surface plasmon resonance (SPR) [25], capillary electrophoresis with laser-induced fluorescence detection [26], and others were also developed to detect SEA [10,18,24,27,28]. The disadvantages of these methods are the extraction and concentration of enterotoxins prior to analysis, time consumption, and personal skills. Immunoassays are commonly used to detect proteins, but the low stability of the antibodies used in the assay often affects the accuracy of the results and requires more analysis time [29,30]. Compared with antibodies, aptamers have many significant advantages, including low immunogenicity, chemical durability, low molecular weight, and high affinity, and they are relatively inexpensive [31]. Therefore, it is necessary to develop an efficient, sensitive, specific, and easy-to-use aptamer-based method to detect SEA.

Recently, aptamer-based fluorescent biosensors received great attention due to numerous advantages, such as high sensitivity [32], specificity [33], and simplicity [34,35,36]. DNA silver nanoclusters (DNA-AgNCs) have the advantages of high quantum yield, strong photostability, low toxicity, adjustable fluorescence emission [37], and excellent biocompatibility [37,38,39,40,41,42]. In this study, we combined the silver salt with aptamers coupled with nucleation sequences at the appropriate temperature and reducing agents to synthesis the DNA-AgNCs as fluorescent luminescent nanomaterials. The aptamer-functionalized DNA-AgNCs with specific recognition of the SEA domain can be formed without purification by using the SEA aptamer as the template [43]. The aptamer can specifically recognize the SEA ligand and bind to its target with high affinity and specificity. DNA-AgNCs can specifically recognize SEA while producing fluorescence, and the DNA sequence does not require any modification to avoid the effect of space resistance [44,45].

Polypyrrole nanoparticles (PPyNPs) have a π-rich structure, and single-stranded DNA (ssDNA) can be adsorbed onto PPyNPs by strong π–π stacking [46,47]. In addition, PPyNPs can quench the fluorescence of near-surface materials. Moreover, PPyNPs also have the properties of selective absorption of DNA and low specific absorption of proteins [47,48,49]. Therefore, we chose the PPyNPs as an effective quenching agent for DNA-AgNCs and combined PPyNPs with DNA-AgNCs to assemble the SEA aptamer sensors. These properties of PPyNPs can not only increase their absorption ability toward DNA-AgNCs, but also reduce their nonspecific binding to SEA. When PPyNPs bind to DNA-AgNCs, the energy of the DNA-AgNC fluorescence region is absorbed by PPyNPs, resulting in fluorescence quenching. After the target SEA is added, the appropriate fragment of SEA and DNA-AgNCs causes the release of the DNA-AgNCs from the PPyNP surface and restores the fluorescence, which is measured and used for quantitation.

## 2. Materials and Methods

### 2.1. Chemicals and Materials

HPLC purified oligonucleotides were obtained from Shanghai Sangon Biotechnology Co., Ltd. (Shanghai, China), and all the sequences are shown in Table S1 (Supplementary Materials). Silver nitrate (99%) and bovine serum albumin (BSA) were purchased from Aladdin Chemistry Co., Ltd. (Shanghai, China). Sodium chloride, disodium hydrogen phosphate, sodium dihydrogen phosphate, citric acid, potassium dihydrogen phosphate, potassium chloride, NaBH_4_, polyvinyl alcohol, polypyrrole, and FeCl_3_·6H_2_O were purchased from Sinopharm Chemical Reagent Co., Ltd. (Shanghai, China). Staphylococcal enterotoxin A (SEA), staphylococcal enterotoxin B (SEB), staphylococcal enterotoxin C1 (SEC1), and staphylococcal enterotoxin D (SED) (99%) were purchased from Beijing Bomai Biotechnology Co., Ltd. (Beijing, China). Ultrapure water (18.2 MΩ/cm) was used in all experiments.

### 2.2. DNA-AgNC/PPyNP Characterization

The ultraviolet–visible light (UV–Vis) absorption spectra were recorded using a UV-1800 spectrophotometer (Shimadzu Co., Kyoto, Japan). The emission spectra were obtained on a Hitachi F-7000 fluorescence spectrophotometer (Hitachi Ltd., Tokyo, Japan). Transmission electron microscopy (TEM) was performed at room temperature using a JEM-2100HR transmission electron microscope (JEOL, Tokyo, Japan) with an accelerating voltage of 200 kV.

### 2.3. Preparation of DNA-AgNCs

The DNA-AgNCs were synthesized according to a previously reported synthesis method with slight modification [50], and the whole reaction was carried out in the dark. Briefly, a 100 μM DNA template was dissolved in phosphate buffer (pH = 7.2), heated at 95 °C for 10 min, and cooled in an ice bath for 10 min. Then, 300 μM of AgNO_3_ was added to the solution, and the mixture was incubated at 4 °C for 30 min. Afterward, freshly prepared NaBH_4_ was immediately added to the resulting mixture and rapidly stirred for 5 min. The DNA-AgNCs were prepared at a molar ratio of 1:20:20 (DNA:Ag^+^:BH_4_^−^). The solution was incubated in the dark at 4 °C for 6 h to obtain the maximum fluorescence intensity (FI). Unless otherwise stated, the concentration of the prepared DNA-AgNCs refers to the final concentration of DNA used.

### 2.4. Preparation of PPyNPs

PPyNPs were synthesized and purified according to previous reports with some modifications [51]. Briefly, 1.3 g of FeCl_3_∙6H_2_O and 1.5 g of polyvinyl alcohol (PVA) were dissolved in 50 mL of deionized water and stirred for 1 h in an ice bath to obtain a pale-yellow solution. Then, after adding 240 μL of pyrrole monomer, the solution was exposed to the oxidant in the solution (FeCl_3_∙6H_2_O) to initiate polymerization. After a few minutes, the solution changed from yellowish to black, and it was then stirred for 4 h. After completion of the reaction, the samples were separated by centrifugation and washed three times with hot water. The samples were dialyzed in the dark using a 25-kDa molecular weight cut-off (MWCO) dialysis bag against ultrapure water for 48 h and replaced every 6 h. PPyNPs were obtained after drying.

### 2.5. Fluorescence Detection of SEA Toxins

The DNA-AgNCs@PPyNPs were prepared as follows: PPyNPs (8 mg/mL) and DNA-AgNCs (2.4 μM) were mixed for 10 min at 37 °C. Then, SEA was added to the DNA-AgNCs@PPyNPs at different concentrations in the range of 0.5 to 1000 ng/mL, and the solutions were incubated for 30 min at 37 °C. The fluorescence emission spectrum was recorded at 634 nm.

### 2.6. Sample Preparation and Measurement

Since dairy products are often contaminated with enterotoxin, milk was selected as the actual sample to evaluate the performance of the biosensors in complex media. Milk samples purchased from supermarkets were used to verify the experimental results. All milk samples were within the shelf life and were not contaminated with large amounts of SEA. A series of SEA standard solutions with a concentration gradient were added to 100 μL milk for standard recovery. Each sample was centrifuged at 4000 rpm for 10 min at 25 °C, and the fat layer was removed. Then, the sample was added to the sensing system, and the FI of the mixed reactant was directly measured at 634 nm on a fluorescence spectrophotometer. Six parallel replicate experiments were performed for each concentration, and the entire process was protected from light.

## 3. Results

### 3.1. Principle of the Detection Method

**Scheme 1:**
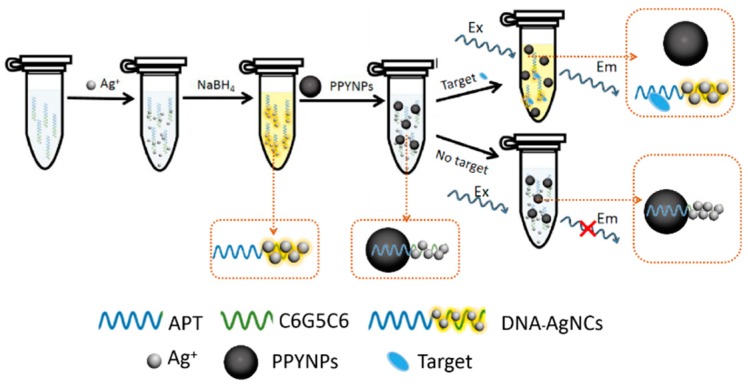
Schematic illustration of the dynamic quenching principle for staphylococcal enterotoxin A (SEA) sensing.

A schematic diagram of the detection methods of SEA is shown in Scheme 1. We optimized the DNA sequence to increase FI to improve sensor sensitivity. The DNA template in DNA-AgNCs consists of two fragments: one is the main luminescent fragment of AgNCs at the 5’ end, which is used to increase the sensitivity by increasing the fluorescence range, and the other is the 3’ end of the SEA aptamer (apt) recognition target segment [36]. DNA-AgNCs can be adsorbed onto PPyNPs by strong π–π stacking interactions, directing energy transfer from AgNCs (energy donors) to PPyNPs (energy receptors). The fluorescence emitted from the luminous region of DNA-AgNCs is absorbed by PPyNPs, which leads to fluorescence quenching. In the presence of the target SEA, the binding of SEA to the aptamer of DNA-AgNCs causes the release of DNA-AgNCs from the surface of PPyNPs, resulting in fluorescence recovery. With the increase in SEA concentration, the FI increases proportionately, allowing SEA detection to be achieved.

### 3.2. Optimization of DNA-AgNC Conditions

**Figure 1:**
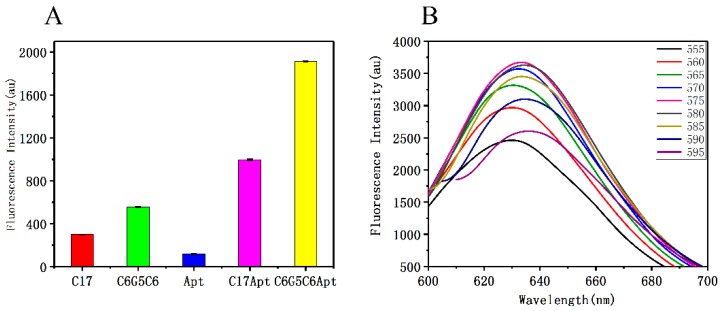
(**A**) The fluorescence intensity (FI) of various DNA sequences. (**B**) The fluorescence emission spectra of C6G5C6-aptamer (apt)-silver nanoclusters (AgNCs) and polypyrrole nanoparticles (PPyNPs) with the λ_ex_ increasing from 555 nm to 595 nm.

Since the method is more sensitive when the FI of DNA-AgNCs is higher, we studied the conditions for the preparation of DNA-AgNCs in order to maximize the fluorescence efficiency. We firstly investigated the effect of the DNA sequence on the fluorescence efficiency of the DNA-AgNCs. Recently, studies showed that Ag^+^ can be selectively bonded to the cytosine [52]. The fluorescence of DNA-AgNCs can be enhanced by inserting guanine into a cytosine-rich fragment. There is no positive/negative interaction between adenine and thymidine and DNA-AgNCs. Guo reported that the sequence with five guanines inserted in cytosine has the strongest fluorescence and is not affected by the insertion position [53]. Therefore, the C6G5C6, C17, C17-apt, apt, and C6G5C6-apt sequences (Table S1, Supplementary Materials; underlined sequence is the aptamer sequence) were used as templates to prepare AgNCs. The fluorescence emission intensity which is shown in Figure 1A (λ_EX_ = 575 nm and λ_Em_ = 634 nm) and Figure S1 (Supplementary Materials) revealed that the FI of the five guanines replacing the five cytosines in the middle of C17 increased 1.85-fold, which confirmed that the previously reported substitution of guanine for cytosine in the fragment could enhance the fluorescence of DNA-AgNCs. In addition, compared to the simple aptamer sequence, the fluorescence of aptamers connected to C17 and C6G5C6 increased 8.40-fold and 16.13-fold, respectively, indicating that the increase in aptamer fluorescence was related to the connected sequence. This may be because, when apt was linked to C6G5C6, apt could effectively stimulate the fluorescence potential of the C6G5C6-templated AgNCs [54]. Therefore, in order to obtain a larger range of FI for detection, the C6G5C6-apt-AgNCs were synthesized using C6G5C6-apt as a template in the subsequent experiments. Therefore, in order to obtain a larger range of FI for detection, the C6G5C6-apt-AgNCs were synthesized using C6G5C6-apt as a template in the subsequent experiments. Furthermore, the secondary structure of C6G5C6-apt (Figure S2, Supplementary Materials) is similar to apt and has lower Gibbs free energy, which is conducive to identifying aptamers.

The fluorescence emission spectra of C6G5C6-apt-AgNCs at different excitation wavelengths are displayed in Figure 1B. These spectra showed that the emissions corresponding to different excitation wavelengths are also slightly different. A possible reason behind the difference in FI may be due to the presence of a non-single number of atoms in synthesized silver nanoclusters. As the number of atoms in different clusters was different, the transition energy was also different, and the cluster distribution of different atoms followed a Poisson distribution, which resulted in the difference in FI spectra correspondence to different excitation [55]. At the excitation wavelength of 575 nm, the emission peak was the highest. Therefore, 575 nm was selected as the excitation wavelength post-detection.

**Figure 2:**
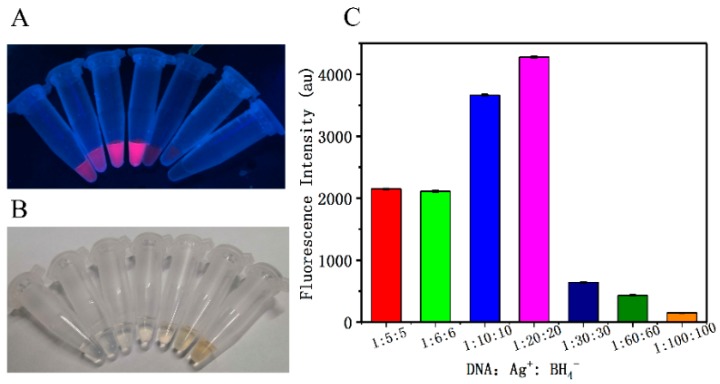
The images of a C6G5C6-apt-AgNC solution acquired under ultraviolet (UV) light (**A**) and under daylight (**B**). (**C**) Effect of the ratio of oligonucleotide (Table S1, Supplementary Materials) to Ag^+^ on the fluorescence spectra of C6G5C6-apt-AgNCs.

**Figure 3:**
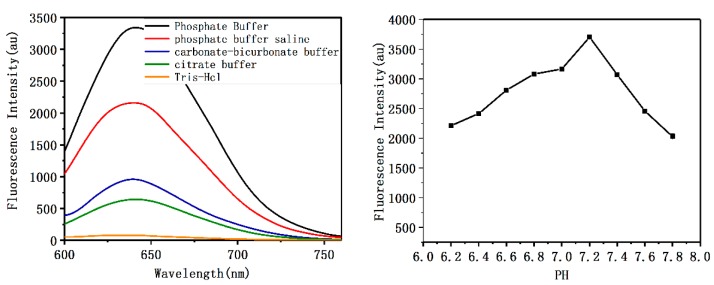
The FI of C6G5C6-apt-AgNCs in buffers at various pH values.

Next, in order to obtain the highest fluorescence intensity, the effect of the molar ratio of DNA:Ag^+^:BH_4_^−^, type of buffer, pH, and incubation time on the fluorescence efficiency of C6G5C6-apt-AgNCs was evaluated. The results showed that the ratio of the oligonucleotide to Ag^+^ is the most important factor affecting the FI of C6G5C6-apt-AgNCs. The effect of different ratios of DNA to Ag^+^ and BH_4_^−^ on the FI is shown in Figure 2. The results revealed that a ratio of below 1: 10 is insufficient to yield the higher production of silver nanocluster. When the ratio exceeds 1: 20, an excessive amount of silver clusters is formed and aggregation occurs, leading to fluorescence self-quenching. Therefore, at a concentration ratio of 1:20, a high quantum yield of AgNCs is easy to achieve. As shown in Figure 3, the highest fluorescence intensity was obtained using the following conditions: 1:20:20 molar ratio of DNA:Ag^+^:BH_4_^−^, phosphate buffer at pH 7.2, and incubation at 4 °C for 6 h, which are ideal for producing brightly emitting DNA-AgNCs and promoting the largest C6G5C6 signal enhancement.

### 3.3. Characterization of AgNCs and PPyNPs

**Figure 4:**
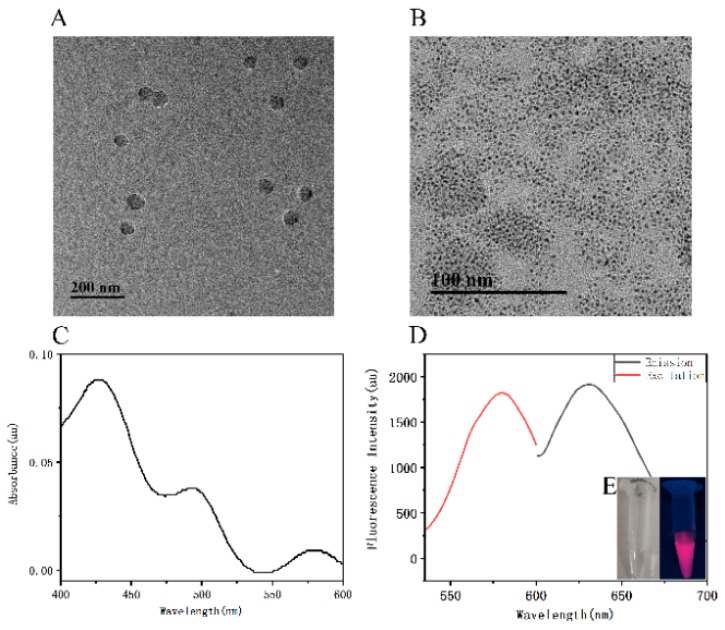
Transmission electron microscopy (TEM) image of C6G5C6-apt-AgNCs (**A**) and PPyNPs (**B**). (**C**) UV–visible light (UV–Vis) absorption spectrum of the C6G5C6-apt-AgNCs. (**D**) The FI spectra of the excitation spectrum (red curve) and emission spectrum (black curve) of the C6G5C6-apt-AgNCs. Inset: the images of a C6G5C6-apt-AgNCs solution acquired under daylight (left) and UV light (right).

The TEM image showed that C6G5C6-apt-AgNCs and PPyNPs were monodispersed and uniform in aqueous solution. It could be observed that the C6G5C6-apt-AgNCs were spherical and uniform in size, at about 3 nm [56]. The UV absorption spectra and the FI spectra of C6G5C6-apt-AgNCs are shown in Figure 4C,D, respectively.

The peaks at 427 nm and 493 nm corresponded to the plasma absorption peaks of silver nanoparticles, and the peak at 579 nm could be attributed to the absorption band of AgNCs, indicating the successful synthesis of C6G5C6-apt-AgNCs [57]. The maximum absorbance of the PPyNPs aqueous solution at 804 nm was 1.483 (Figure S3, Supplementary Materials). In addition, the spectra also revealed that the aqueous solution had a very strong wide absorption in the near-infrared region, and the wide characteristic absorption peak from the visible region to the near-infrared region corresponded to the doping polypyrrole of the bipolar metal state. The strong near-infrared light absorption properties also confirmed that PPyNPs could be used for fluorescence quenching of C6G5C6-apt-AgNCs.

### 3.4. Optimized Detection Conditions

**Figure 5:**
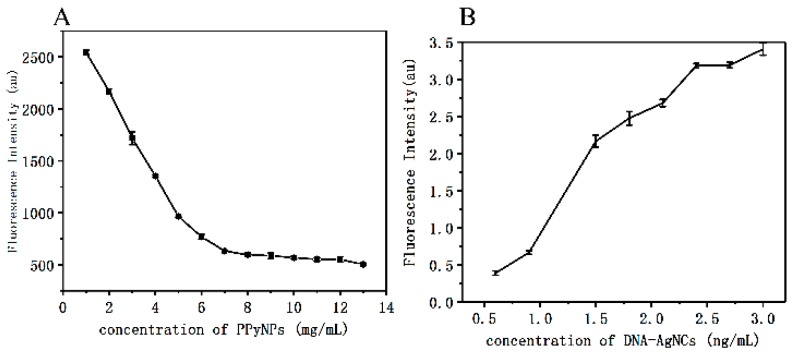
(**A**) The FI of C6G5C6-apt-AgNCs in the presence of various concentrations of PPyNPs (1, 2, 3, 4, 5, 6, 7, 8, 9, 10, 11, 12, and 13 mg/mL). (**B**) The FI of different concentrations of C6G5C6-apt-AgNCs at 634 nm in the presence of PPyNPs (8 mg/mL) and SEA (1 μg/mL).

Fluorescence quenching of C6G5C6-apt-AgNCs induced by PPyNPs at room temperature was studied under optimal reaction conditions. After mixing C6G5C6-apt-AgNCs and PPyNPs, the fluorescence decreased rapidly. We set a 10-min interval to allow the fluorescence to be completely quenched. The fluorescence signal changes of a fixed amount of C6G5C6-apt-AgNCs combined with different concentrations of PPyNPs (1, 2, 3, 4, 5, 6, 7, 8, 9, 10, 11, 12, and 13 mg/mL) are shown in Figure 5A.

When 8 mg/mL PPyNPs or more were added, the fluorescence remained basically unchanged. Therefore, 8 mg/mL was selected as the optimal concentration for fluorescence quenching, at which the quenching efficiency was the highest. At the same time, we also studied the recovery by different concentrations of C6G5C6-apt-AgNCs (0, 0.3, 0.6, 0.9, 1.2, 1.5, 1.8, 2.1, 2.4, 2.7, and 3 μM) with a fixed concentration of 1 μg/mL of SEA at the best quenching concentration of PPyNPs (8 mg/mL). With the increase in C6G5C6-apt-AgNCs concentration, the fluorescence recovered gradually until the fluorescence reached its maximum at 2.4 μM, and then remained stable.

### 3.5. Feasibility Analysis

**Figure 6:**
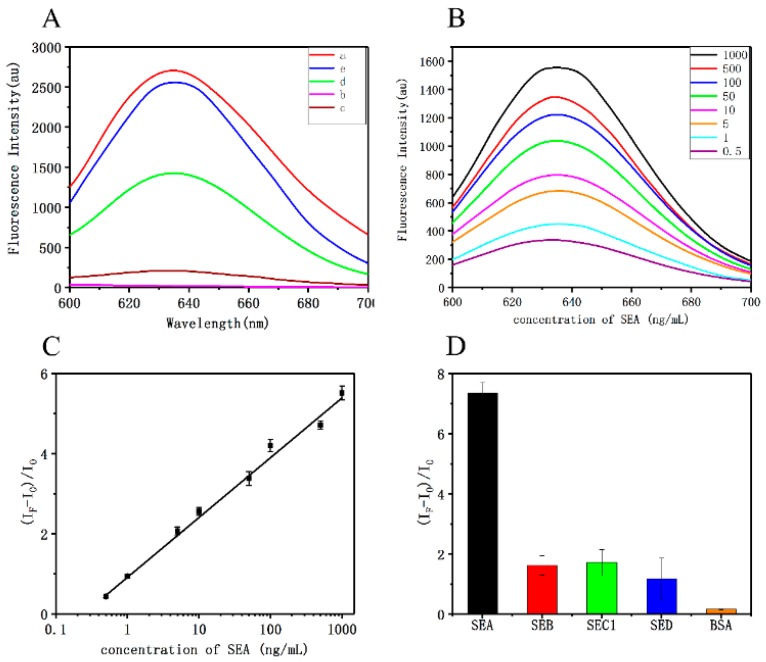
(**A**) Fluorescence emission spectra of C6G5C6-apt-AgNCs under various conditions: (a) C6G5C6-apt-AgNCs (b) PPyNPs (c) C6G5C6-apt-AgNCs + PPyNPs (d) C6G5C6-apt-AgNCs + PPyNPs + SEA (e) C6G5C6-apt-AgNCs + SEA (C6G5C6-apt-AgNCs, 2.4 μM; PPyNPs, 8 mg/mL; SEA, 500 ng/mL). (**B**) Fluorescence spectra in the presence of increasing concentration of the target. (**C**) A linear relationship is obtained when the concentration of SEA is in the range of 0.5–1000 ng/mL. (**D**) Relative FI of the proposed bioassay for SEA and other toxins (SEB, SEC1, SED, bovine serum albumin (BSA)).

In order to demonstrate the feasibility of the proposed method, the feasibility of quantitative detection of SEA by C6G5C6-apt-AgNCs was assessed. The fluorescence spectra were recorded under different conditions. The strong emission peak of C6G5C6-apt-AgNCs appeared at 634 nm (curve a, Figure 6A), and the addition of PPyNPs resulted in fluorescence quenching, which reduced the FI of this peak (curve c, Figure 6A). After adding SEA, the fluorescence signal recovered rapidly (curve d, Figure 6A) due to the release of C6G5C6-apt-AgNCs from C6G5C6-apt-AgNCs@PPyNPs caused by the binding of SEA to apt in C6G5C6-apt-AgNCs. Furthermore, the addition of the target without PPyNPs did not significantly affect the FI (curve b, Figure 6A), indicating that there was no interaction between the target itself and the C6G5C6-apt-AgNCs. These results demonstrate the feasibility of the detection of SEA with a fluorescent aptamer.

### 3.6. Analytical Performance

Under the selected conditions, the fluorescence spectra of the samples with different concentrations of SEA were recorded. The spectra displayed in Figure 6C show that the FI of DNA-AgNCs increased proportionately with the increase in SEA concentration in the range of 0.5 to 1000 ng/mL (*Y* = 1.4917*X* + 0.9100, *R*^2^ = 0.9948), and the limit of detection (LOD) was 0.3393 ng/mL. The detection limit was obtained from the calculation of 3 σ/K, where σ is the standard deviation calculated from three parallel blank signals, and K is the slope of the calibration curve. Compared with the previously published detection methods, the sensor developed here has a relatively good test performance and can be used for sensitive detection of SEA.

In order to evaluate the specificity of the system for detecting SEA, three other *S. aureus* enterotoxins (SEB, SEC1, SED) and BSA at the same concentration as SEA (1 µg/mL) were added to the detection system under optimal conditions, and the fluorescence analysis was performed. The FI of BSA, SEB, SEC1, and SED was much lower than that of SEA, which may be due to the specific affinity of the SEA aptamer for the SEA toxin. The results showed that the detection system has excellent selectivity for SEA.

### 3.7. Detection of SEA in Milk Samples

In order to evaluate the feasibility of the sensor to detect SEA in an actual sample, milk was selected as the real sample. Three different concentrations of SEA (six replicates) were added to the milk samples for recovery experiments to determine the recovery level of the test method. The results are summarized in Table 1, and the recovery was found to be between 87.70% and 94.65%. Therefore, the results of the testing of the developed method revealed the high selectivity, sensitivity, and stability of this method for the analysis of SEA in real samples.

## 4. Conclusions

In this study, we developed a new fluorescent AgNCs sensing system based on the use of an aptamer as a template. The fluorescence decreased when the method took advantage of the high fluorescence quenching efficiency of PPyNPs, which, combined with C6G5C6-apt-AgNCs, decreased the fluorescence emission and allowed the detection of the SEA content by the SEA concentration-dependent fluorescence recovery. Depending on the affinity and specificity of the aptamers, the detection method showed high sensitivity and good specificity with an LOD of 0.3393 ng/mL, and a linear range from 0.5 to 1000 ng/mL (*Y* = 1.4917*X* + 0.9100, *R*^2^ = 0.9948). An excellent recovery rate was achieved for detecting SEA in the real milk sample, confirming the feasibility and reliability of this method using the aptamer sensor. The method has a short detection time and simple operation. Basically, no pretreatment is required for samples, showing comparable results to previous detection methods. One of the drawbacks of the methodology developed is the need for the test to be performed in the dark. Conclusively, this fluorescent biosensor can be developed as a potential universal detection system, which can directly detect other targets of interest by simply changing the corresponding adaptor of the detection object.

## Figures and Tables

**Table 1:** The recovery results for staphylococcal enterotoxin A (SEA) detection in milk.

Sample	Added (ng/mL)	Detection (ng/mL) (Mean ± SD, n = 6)	Recovery Ratio (%)
Sample 1	50	47.3291 ± 3.0103	94.65%
Sample 2	100	87.7010 ± 5.6440	87.70%
Sample 3	150	138.4932 ± 5.2269	92.33%

## Supplementary Materials

The following are available online at https://www.mdpi.com/2073-4360/12/1/152/s1: Table S1: Oligonucleotides names, abbreviations, and DNA sequences; Figure S1: AgNCs synthesized with different nucleotides sequences (Table S1) as the template; Figure S2: The secondary structure of Apt(A), C6G5C6-apt(B), and C17-apt(C); Figure S3: UV–Vis absorption spectrum of the PPyNPs.
