# Supplementary material for: A Label-Free Fluorescent Aptasensor for Detection of Staphylococcal Enterotoxin A Based on Aptamer-Functionalized Silver Nanoclusters
Source: Polymers (Basel). 2020 Jan 7;12(1):152. doi: 10.3390/polym12010152 (PMC7023026; doi:10.3390/polym12010152)
Supplement: Supplementary file 1 [file polymers-12-00152-s001.pdf]

Table S1. Oligonucleotides names, abbreviations and DNA sequences

Name	Abbreviations	Sequences (5′→3′)
C17	C17	CCCCCCCCCCCCCCCCC
C6G5C6	C6G5C6	CCCCCCGGGGGCCCCCC
SEA aptamer	Apt	AGCAGCACAGAGGTCAGATGTACTTATGCATTTCCTCCCACGATCTTATTTGAGAGTGACCCTATGCGTGCTACCGTGAA
C17-aptamer	C17-apt	CCCCCCCCCCCCCCCCCAGCAGCACAGAGGTCAGATGTACTTATGCATTTCCTCCCACGATCTTATTTGAGAGTGACCCTATGCGTGCTACCGTGAA
C6G5C6-aptamer	C6G5C6-apt	CCCCCCGGGGGCCCCCCAGCAGCACAGAGGTCAGATGTACTTATGCATTTCCTCCCACGATCTTATTTGAGAGTGACCCTATGCGTGCTACCGTGAA

Table S2. Comparison of the performance of the developed bioassay detected SEA with other methods.

Method Strategy	Detection	LOD（ng ml-1）	Linear range	Ref.
quartz crystal microbalance	antibody	1 ng /mL	50-1000 ng/mL	[1]
electrochemical immunosensor	antibody	33.9 ng/mL	0.016-0.150 mg/mL	[2]
DAS-ELISA	antibody	32 pg/mL	4-1000 pg/mL	[3]
chemiluminescence enzyme immunoassay	antibody	3.2 pg/mL	6.4 pg/mL-1600 pg/mL	[4]
surface plasmon resonance	antibody	100 ng / mL	100-1000 ng/mL	[5]
LC-ESI / MS		0.5 ng/mL	1-100 ng/mL	[6]
capillary electrophoresis with laser-induced fluorescence detection	antibody	3 aM	0.3 nM and 6.5 nM	[7]
evanescent wave biosensor	antibody	10 ng/mL	10–100 ng/mL	[8]
DNA-AgNCs-PPYNPs	aptamer	0.3393 ng/mL	0.5 to 1000 ng/mL	Present work

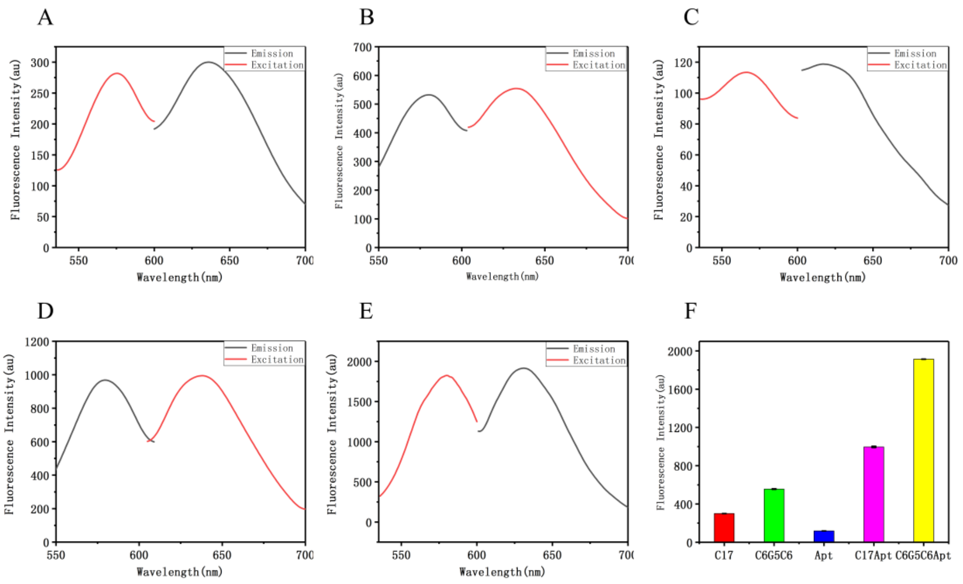

Figure S1. AgNCs synthesized with different nucleotides sequences (Table S1) as the template.

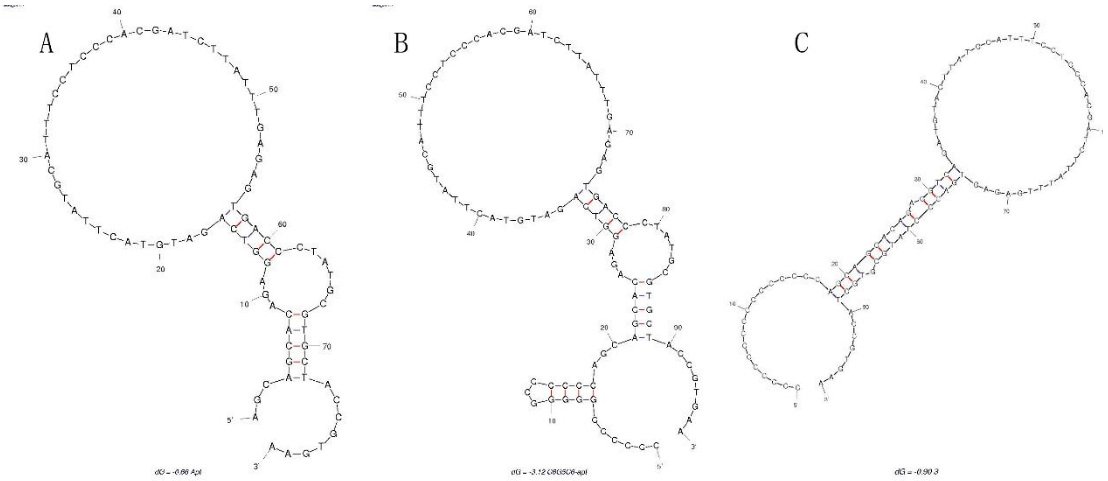

Figure S2. The secondary structure of Apt(**A**), C6G5C6-apt(**B**) and C17-apt(**C**)

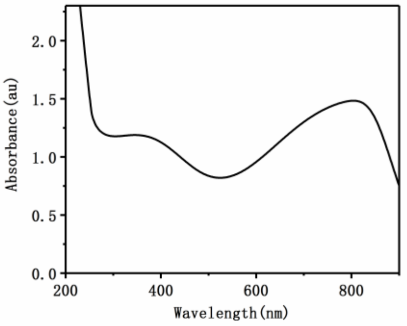

Figure S3. UV–vis absorption spectrum of the PPyNPs

1. Ben Haddada, M.; Salmain, M.; Boujday, S. Gold colloid-nanostructured surfaces for enhanced piezoelectric immunosensing of staphylococcal enterotoxin A. *Sensors and Actuators B: Chemical* **2018**, *255*, 1604–1613, doi:https://doi.org/10.1016/j.snb.2017.08.180.
2. Pimenta-Martins, M.G.R.; Furtado, R.F.; Heneine, L.G.D.; Dias, R.S.; Borges, M.d.F.; Alves, C.R. Development of an amperometric immunosensor for detection of staphylococcal enterotoxin type A in cheese. *Journal of Microbiological Methods* **2012**, *91*, 138–143, doi:https://doi.org/10.1016/j.mimet.2012.05.016.
3. Clarisse, T.; Michèle, S.; Olivier, T.; Valérie, E.; Vincent, L.M.; Jacques-Antoine, H.; Michel, G.; Florence, V. Detection and quantification of staphylococcal enterotoxin A in foods with specific and sensitive polyclonal antibodies. *Food Control* **2013**, *32*, 255–261, doi:https://doi.org/10.1016/j.foodcont.2012.11.021.
4. Zhang, C.; Liu, Z.; Li, Y.; Li, Q.; Song, C.; Xu, Z.; Zhang, Y.; Zhang, Y.; Ma, Y.; Sun, Y., et al. High sensitivity chemiluminescence enzyme immunoassay for detecting staphylococcal enterotoxin A in multi-matrices. *Analytica Chimica Acta* **2013**, *796*, 14–19, doi:https://doi.org/10.1016/j.aca.2013.07.044.
5. Tsai, W.-C.; Li, I.-C. SPR-based immunosensor for determining staphylococcal enterotoxin A. *Sensors and Actuators B: Chemical* **2009**, *136*, 8–12, doi:https://doi.org/10.1016/j.snb.2008.10.061.
6. Andjelkovic, M.; Tsilia, V.; Rajkovic, A.; De Cremer, K.; Van Loco, J. Application of LC-MS/MS MRM to Determine Staphylococcal Enterotoxins (SEB and SEA) in Milk. *Toxins* **2016**, *8*, doi:10.3390/toxins8040118.
7. Lam, M.T.; Wan, Q.H.; Boulet, C.A.; Le, X.C. Competitive immunoassay for staphylococcal enterotoxin A using capillary electrophoresis with laser-induced fluorescence detection. *Journal of Chromatography A* **1999**, *853*, 545–553, doi:https://doi.org/10.1016/S0021-9673(99)00677-9.
8. Rasooly, L.; Rasooly, A. Real time biosensor analysis of Staphylococcal enterotoxin A in food. *International Journal of Food Microbiology* **1999**, *49*, 119–127, doi:https://doi.org/10.1016/S0168-1605(99)00053-7.
